# Influence of Body Mass Index on the Evaluation and Management of Pediatric Abdominal Pain in the Emergency Department

**DOI:** 10.5811/westjem.59287

**Published:** 2023-08-08

**Authors:** Carly A. Theiler, Morgan Bobb Swanson, Ryan Squires, Karisa K. Harland

**Affiliations:** * University of Iowa, Department of Emergency Medicine, Iowa City, Iowa; † Indiana University, Department of Emergency Medicine, Bloomington, Indiana

## Abstract

**Introduction:**

Childhood obesity is a serious concern in the United States, with over one third of the pediatric population classified as obese. Abdominal pain is one of the most common chief complaints among pediatric emergency department (ED) visits. We hypothesized that overweight and obese children being evaluated in the ED for abdominal pain would have higher resource utilization than their normal and underweight peers.

**Methods:**

This was a retrospective review of pediatric patients <18 years who presented with abdominal pain to the ED of a tertiary care center from January 1, 2014–September 3, 2020. Patients were excluded if they did not have both a height and weight recorded. We categorized patients as underweight (body mass index [BMI] <5^th^ percentile); normal weight (BMI 5^th^ to <85^th^ percentile), overweight (BMI 85^th^ to <95^th^ percentile); or obese (BMI ≥95^th^ percentile). Descriptive statistics were used to examine the study population. We used chi-square tests to examine the differences in patient characteristics between normal/underweight patients and overweight/obese patients. The Kruskal-Wallis test was completed for examining differences in the medians. We used multivariable logistic regression to examine visit characteristics associated with overweight/obese patients, including ED interventions, testing, and length of stay (LOS).

**Results:**

Of the 184 subjects included in the analysis, nine (4.9%) were underweight, 108 (58.7%) were normal weight, 21 (11.4%) were overweight, and 46 (25.0%) were obese. Patients with a BMI of ≥85^th^ percentile were older (median 15 vs 13 years, *P* = 0.01). They were otherwise similar in demographics. There was no significant difference between normal/underweight and overweight/obese subjects in disposition (37% vs 43% discharge, *P* = 0.38), 72-hour return (7% vs 6%, *P* = 0.82), ED LOS (median 4.42 vs 3.95 hours, *P* = 0.195), or inpatient LOS (median 42.0 vs 34.2 hours, *P* = 0.06). There were no statistically significant differences in total number of ED tests or interventions received by overweight/obese patients compared to normal/underweight patients, and each subject received a median of six tests (interquartile range [IQR] 4–7) and two interventions (IQR 1–3).

**Conclusion:**

Among pediatric patients presenting to the ED with abdominal pain, we found that patient characteristics and ED resource utilization (including testing, intervention, disposition, and LOS) did not differ significantly across BMI categories.

## INTRODUCTION

Childhood obesity is a serious concern in the United States, with over one third of the pediatric population classified as overweight/obese.^1^^,^^2^ A body mass index (BMI) elevated above normal has been associated with numerous illnesses in childhood and higher healthcare utilization/expenditures, including increased outpatient visits, prescription drug use, and emergency department (ED) visits, as well as a higher likelihood of hospital admission.^3^^,^^4^ In addition, overweight and obese children are more likely than their normal-weight peers to receive their routine medical care in an ED rather than a primary care setting.^5^

Abdominal pain is a common chief complaint among pediatric ED visits.^6^ Patients with an elevated BMI have been found to have higher rates of functional gastrointestinal (GI) disorders including constipation, gastroesophageal reflux disease, irritable bowel syndrome, encopresis, and functional pain.^7^^,^^8^ Obese pediatric patients also have higher rates of persistent pain with functional GI disorders, and higher rates of overall pain reporting in the outpatient setting.^9^^,^^10^ Obesity may also limit the accuracy of a physical exam and is a comorbidity that may contribute to missed or delayed diagnosis.^11^^,^^12^ In addition, in children who present to the ED with appendicitis, there is increased likelihood of non-diagnostic ultrasound and need for further imaging in obese children.^13^^,^^14^

We hypothesized that overweight and obese children being evaluated in the ED for abdominal pain would have higher resource utilization than their normal and underweight peers. To date, no study to our knowledge has examined these factors in the pediatric abdominal pain population.

## METHODS

This was a retrospective review of pediatric patients seen in the ED at a midwestern academic center with a freestanding tertiary children’s hospital, with an annual pediatric ED census of approximately 15,000 patients. This was an institutional review board-approved study. We abstracted data from the electronic health record (EHR) for ED visits between September 1, 2014–September 3, 2020. Patients were included who were <18 years of age and presented to the ED with a chief complaint of abdominal pain. Because both height and weight are required to calculate BMI, we excluded patients if they did not have a recent height recorded in the EHR. Once patients were identified using this simple criterion, a single physician completed templated chart abstraction and recorded objective demographic and visit-level data directly into a secure RedCap database hosted at University of Iowa.^1^^,^^2^ REDCap (Research Electronic Data Capture) is a secure, web-based software platform designed to support data capture for research studies. A second blinded abstractor randomly reviewed 10% of charts to ensure interrater reliability; there were no discrepancies.

Pediatric BMI cutoffs are defined using a BMI-for-age percentile; therefore, we calculated BMI as a percentile using the US Centers for Disease Control and Prevention age-based calculator.^15^ Patients were categorized as underweight (BMI <5^th^ percentile); normal weight (BMI 5^th^ to <85^th^ percentile); overweight (BMI 85^th^ to <95^th^ percentile), or obese (BMI ≥95^th^ percentile). We defined ED interventions as pediatric gastroenterology consult, pediatric surgery consult, or administration of antiemetic, intravenous fluids, or pain medications, and we defined ED testing as bloodwork (basic metabolic panel, liver function test, lipase, complete blood count, C-reactive protein, erythrocyte sedimentation rate), enteric panel, urinalysis or urine culture, abdominal radiograph, abdominal ultrasound, or other advanced imaging (computed tomography abdomen or magnetic resonance imaging abdomen).

Descriptive statistics were used to examine the study population, and we used chi-square tests to examine the differences in patient characteristics between normal/underweight patients (BMI <85^th^ percentile) and overweight/obese patients (BMI ≥85^th^ percentile), with *P* < 0.05 considered statistically significant. The Kruskal-Wallis test was completed for examining differences in the medians. We used multivariable logistic regression to examine visit characteristics associated with overweight/obese patients, controlling for age. All statistical analyses were performed using Statistical Analysis Software version 9.3 (SAS Institute Inc, Cary, NC).

## RESULTS

We included a total of 184 patient encounters in the analysis. Of the 184 subjects, nine (4.9%) were underweight (BMI <5^th^ percentile), 108 (58.7%) were normal weight (BMI 5^th^ to <85^th^ percentile); 21 (11.4%) were overweight (BMI 85^th^ to <95^th^ percentile); and 46 (25.0%) were obese (BMI ≥95^th^ percentile). Demographic characteristics of the study population are shown in Table 1. Patients with a BMI of ≥85^th^ percentile were older (median 15 years vs 13 years, *P* = 0.01) but were otherwise similar in demographics including gender, race, and triage level.

**Table 1.** Characteristics of study population.

Variable	Level	N	Overall N = 184	BMI <85% percentilen = 117	BMI ≥85% percentilen = 67	*P*-Value	Metrics
Age (years)		184	13.64 (9.63, 16.44)	13.02 (8.5, 15.87)	15.08 (11.67, 16.7)	0.01	Median (Q1, Q3)
	13–18	184	103 (55.98%)	59 (50.43%)	44 (65.67%)	0.13	N (%)
	2–6		27 (14.67%)	19 (16.24%)	8 (11.94%)		N (%)
	7–12		54 (29.35%)	39 (33.33%)	15 (22.39%)		N (%)
BMI		184	73 (39, 94.5)	47 (26, 72)	96 (94, 99)	<.001	Median (Q1, Q3)
Height (cm)		184	157.5 (134, 167.6)	152.4 (130, 165.1)	161.3 (149.9, 167.6)	0.02	Median (Q1, Q3)
Weight (kg)		184	52.55 (30.1, 70.25)	46.7 (25, 56.4)	75.3 (59.5, 93.2)	<.001	Median (Q1, Q3)
Gender	Female	184	109 (59.24%)	68 (58.12%)	41 (61.19%)	0.68	N (%)
	Male		75 (40.76%)	49 (41.88%)	26 (38.81%)		N (%)
Race	Asian	184	4 (2.17%)	3 (2.56%)	1 (1.49%)	0.31	N (%)
	Black or African Am		16 (8.70%)	7 (5.98%)	9 (13.43%)		N (%)
	Missing		22 (11.96%)	13 (11.11%)	9 (13.43%)		N (%)
	White		142 (77.17%)	94 (80.34%)	48 (71.64%)		N (%)
Mode of transfer	Air ambulance	63	2 (3.17%)	1 (2.44%)	1 (4.55%)	0.89	N (%)
	Ground ambulance		50 (79.37%)	33 (80.49%)	17 (77.27%)		N (%)
	Private vehicle		11 (17.46%)	7 (17.07%)	4 (18.18%)		N (%)
Mode of arrival	Ambulance	183	6 (3.28%)	3 (2.59%)	3 (4.48%)	0.76	N (%)
	Private vehicle/Public transportation		114 (62.30%)	72 (62.07%)	42 (62.69%)		N (%)
	Transfer from another facility		63 (34.43%)	41 (35.34%)	22 (32.84%)		N (%)
Triage level	Emergent	184	28 (15.22%)	20 (17.09%)	8 (11.94%)	0.35	N (%)
	Non-urgent		2 (1.09%)	2 (1.71%)	0 (0.00%)		N (%)
	Urgent		154 (83.70%)	95 (81.20%)	59 (88.06%)		N (%)

*BMI*, body mass index; *cm*, centimeter; *kg*, kilogram; *Am*, American.

Table 2 shows visit characteristics including ED interventions and testing, with adjusted odds of these after controlling for age. Overall, 112 patients (61%) were admitted to inpatient from the ED, and 12 patients (7%) returned to the ED within 72 hours. There was no significant difference between normal/underweight and overweight/obese subjects in disposition (37 vs 43% discharge from the ED, *P* = 0.38) or 72-hour return rates (7 vs 6%, *P* = 0.82). The overall median ED LOS was 4.09 hours (IQR 3.08–5.53), with no significant difference between normal/underweight and overweight/obese subjects (median 4.42 vs 3.95 hrs, *P* = 0.20). Among the 112 patients who were admitted, the median inpatient LOS was 37.7 hrs (20.9–76.7 hrs), with no significant difference between normal/underweight and overweight/obese subjects (median 42.0 vs 34.2 hrs, *P* = 0.06).

**Table 2.** Visit characteristics.

Variable	Level	Overall N = 184	BMI <85% percentile n = 117	BMI ≥85% percentile n = 67	P-Value	aOR (95% CI)^1^
Disposition	Admit	112 (60.87%)	74 (63.25%)	38 (56.72%)	0.38	1.02 (0.52, 1.97)
	DC to home	72 (39.13%)	43 (36.75%)	29 (43.28%)		1.00 (ref)
72-hour return	N	172 (93.48%)	109 (93.16%)	63 (94.03%)	0.82	1.00 (ref)
	Y	12 (6.52%)	8 (6.84%)	4 (5.97%)		0.91 (0.26, 3.21)
ED LOS (hrs)		4.09 (3.08, 5.53)	4.42 (3.33, 5.57)	3.95 (2.58, 5.2)	0.20	−0.45 (−1.17, 0.28)^2^
Inpt LOS (hrs)		37.7 (20.9, 76.7)	42.0 (26.7, 88.2)	34.2 (18.9, 44.1)	0.06	−7.2 (−23.6, 9.2)^2^
Intervention – Antiemetics	Y	88 (47.83%)	59 (50.43%)	29 (43.28%)	0.35	0.77 (0.42, 1.42)
	N	96 (52.17%)	58 (49.57%)	38 (56.72%)		1.00 (ref)
Intervention – Ped GI	Y	12 (6.52%)	7 (5.98%)	5 (7.46%)	0.70	1.39 (0.41, 4.70)
	N	172 (93.48%)	110 (94.02%)	62 (92.54%)		1.00 (ref)
Intervention – Ped surgery	Y	79 (42.93%)	57 (48.72%)	22 (32.84%)	0.04	0.58 (0.31–1.11)
	N	105 (57.07%)	60 (51.28%)	45 (67.16%)		1.00 (ref)
Intervention – IV fluids	Y	128 (69.57%)	84 (71.79%)	44 (65.67%)	0.39	0.90 (0.46, 1.75)
	N	56 (30.43%)	33 (28.21%)	23 (34.33%)		1.00 (ref)
Intervention – Pain meds	Y	59 (32.07%)	37 (31.62%)	22 (32.84%)	0.87	1.04 (0.54, 2.01)
	N	125 (67.93%)	80 (68.38%)	45 (67.16%)		1.00 (ref)
Any bloodwork	N	18 (9.78%)	15 (12.82%)	3 (4.48%)	0.07	1.00 (ref)
	Y	166 (90.22%)	102 (87.18%)	64 (95.52%)		3.48 (0.95, 12.7)
Enteric panel	Y	16 (8.70%)	8 (6.84%)	8 (11.94%)	0.24	2.17 (0.75, 6.27)
	N	168 (91.30%)	109 (93.16%)	59 (88.06%)		1.00 (ref)
Urinalysis/Urine culture	Y	143 (77.72%)	90 (76.92%)	53 (79.10%)	0.73	0.95 (0.45, 2.02)
	N	41 (22.28%)	27 (23.08%)	14 (20.90%)		1.00 (ref)
Abdominal radiograph	Y	60 (32.61%)	45 (38.46%)	15 (22.39%)	0.03	0.51 (0.26, 1.03)
	N	124 (67.39%)	72 (61.54%)	52 (77.61%)		1.00 (ref)
Any abdominal ultrasound	N	96 (52.17%)	64 (54.70%)	32 (47.76%)	0.37	1.00 (ref)
	Y	88 (47.83%)	53 (45.30%)	35 (52.24%)		1.44 (0.78, 2.66)
Any advanced imaging	N	131 (71.20%)	82 (70.09%)	49 (73.13%)	0.66	1.00 (ref)
	Y	53 (28.80%)	35 (29.91%)	18 (26.87%)		0.95 (0.48, 1.88)
Any lab	N	6 (3.26%)	5 (4.27%)	1 (1.49%)	0.31	1.00 (ref)
	Y	178 (96.74%)	112 (95.73%)	66 (98.51%)		2.27 (0.25, 20.5)
Any imaging	N	46 (25.00%)	24 (20.51%)	22 (32.84%)	0.06	1.00 (ref)
	Y	138 (75.00%)	93 (79.49%)	45 (67.16%)		0.53 (0.26, 1.05)
Number of ED interventions		2 (1, 3)	2 (1, 3)	2 (1, 3)	0.20	0.00 (−0.54, 0.54)^2^
Number of ED tests		6 (4, 7)	6 (4, 7)	6 (5, 7)	0.62	0.00 (−0.45, 0.45)^2^

1 Adjusted for age of patient as a continuous variable.

2 The median difference for those with a BMI in the ≥85% percentile vs the <85% percentile.

*BMI*, body mass index; *aOR*, adjusted odds ratio; *DC,* discharge; *ED*, emergency department; *LOS*, length of stay; *Inpt*, inpatient; *hrs*, hours; *Ped*, pediatric; *GI*, gastroenterology; *IV*, intravenous.

There was no statistically significant difference in total numbers of ED tests or interventions received by overweight/obese patients compared to normal/underweight patients. Each subject received a median of six ED tests (IQR 4–7) and two ED interventions (IQR 1–3). There was no difference in the number of ED interventions (*P* = 0.20) or ED tests (*P* = 0.618) by BMI group. On bivariate analysis, overweight/obese subjects were less likely than normal/underweight subjects to have an abdominal radiograph (22 vs 38%, *P* = 0.03) and less likely to have a pediatric surgery consult (33 vs 49%, *P* = 0.04); however, after controlling for age, this finding was not found to be statistically significant for either abdominal radiograph (aOR 0.51, 95% confidence interval [CI] 0.26–1.03) or pediatric surgery consult (aOR 0.58, 95% CI 0.31–1.11). There were no significant differences in the rate of other individual tests or interventions between the two groups.

## DISCUSSION

Our results did not show a significant difference in ED resource use (testing or intervention) or LOS for overweight/obese pediatric patients presenting to the ED with abdominal pain compared to normal/underweight patients. We analyzed 184 pediatric patients presenting to the ED with a chief complaint of abdominal pain. Of these, 4.9% were underweight, 58.7% normal weight, 11.4% overweight, and 25.0% were obese. Patients with a BMI of ≥85^th^ percentile were older (median 15 years vs 13 years, *P* = 0.01) but otherwise similar in demographics.

There was no significant difference between normal/underweight and overweight/obese subjects in disposition (37 vs 43% discharge from the ED, *P* = 0.38); 72-hour return (7 vs 6%, *P* = 0.82), ED LOS (median 4.42 vs 3.95 hrs, *P* = 0.20), or inpatient LOS (median 42.0 vs 34.2 hrs, *P* = 0.06). There was no statistically significant difference in total number of ED tests or interventions received by overweight/obese patients compared to normal/underweight patients.

The lack of statistical differences in ED resource use and LOS across BMI groups in our population was surprising, as our hypothesis—pediatric patients with elevated BMI who present to the ED with abdominal pain would have higher resource utilization—was based on prior studies demonstrating higher ED utilization and higher rates of functional abdominal pain in overweight and obese pediatric patients.^3^^–^^5^^,^^7^^–^^9^ This lack of statistical difference between BMI groups in regard to ED resource use for abdominal pain could potentially be explained by the general tendency to very closely evaluate and observe all pediatric abdominal pain in the ED, particularly when considering the higher rates of atypical presentations of acute appendicitis or missed diagnoses in the younger population.^16^ Interestingly, our findings demonstrating lack of difference in ED resource utilization across BMI groups in the pediatric abdominal pain population parallel the findings of similar adult studies.^11^^,^^17^

## LIMITATIONS

We were able to analyze only subjects whose BMI could be calculated (both height and weight recorded in the EHR). Unfortunately, height is not routinely obtained in the ED, and in this retrospective review we likely missed a large number of patients due to no height being recorded, resulting in a limited sample size. Notably, we did find that the majority of patients with height and weight recorded also had a primary care physician (PCP) at the same tertiary care institution where the study took place. It could be hypothesized that the “missed population” (patients who did not have a height recorded in the EHR) may have represented a larger proportion of patients who either resided in a more rural area with a PCP outside the tertiary system’s EHR, or who did not have a PCP at all. In a sub-sample of pediatric patients with abdominal pain identified during the study time, those with height and weight recorded in the EHR were older than those who did not have height and weight recorded in the EHR, suggesting that the “missed population” may have had additional characteristics that differed from the study population. Future prospective studies could be aimed at obtaining a height and weight on all patients to eliminate this selection bias.

## CONCLUSION

We found that the ED evaluation of pediatric patients with a chief complaint of abdominal pain did not differ significantly based on body mass index in regard to resource utilization, including testing, intervention, and length of stay. Further research is warranted to assess whether this finding is replicated outside a pediatric tertiary care center, in the general pediatric population.
